# A modified stereotactic frame as an instrument holder for frameless stereotaxis: Technical note

**DOI:** 10.4103/2152-7806.70957

**Published:** 2010-10-11

**Authors:** Arun Angelo Patil

**Affiliations:** Division of Neurosurgery, University of Nebraska Medical Center, 982035 Nebraska Medical Center, Omaha, NE 68198-2035, USA

**Keywords:** Coordinates adjuster, frameless stereotaxis, instrument holder, stereotactic frame

## Abstract

**Background:**

In order to improve the targeting capability and trajectory planning and provide a more secure probe-holding system, a simple method to use a stereotactic frame as an instrument holder for the frameless stereotactic system was devised.

**Methods:**

A modified stereotactic frame and BrainLab vector vision neuronavigation sys¬tem were used together. The patient was placed in the stereotactic head-holder to which a reference array of the neuronavigation system was attached. The pointer of the frameless system was placed in the probe-holder of the frame. An offset in distances was kept between the radius of the arch of the frame and the tip of the pointer so that the pointer was always outside the head during navigation. The offset correction was made on the BrainLab monitor so that the center of the arc of the frame was at the tip of the probe line on the monitor. Then, using the frame’s coordinate adjuster system, the center of the arc was positioned on the target. This method was used to insert depth electrodes (seven procedures) and gain access to the temporal horn (three procedures).

**Results:**

Post-operative scans showed that the accuracy was within 2.5 mm in all three planes for depth electrode placement, and easy access to the temporal horn was obtained in two other patients.

**Conclusion:**

This is a simple method to use a stereotactic frame to improve coordinate and trajectory adjustments and provides a better method to stabilize the pointer and the probe-holder during frameless stereotactic procedures.

## INTRODUCTION

Horsley first introduced frame-based stereotaxis into neurosurgery.[8] Initially, stereotactic systems were used mainly for functional procedures that required accurate placement of probes into deep brain targets. After the introduction of computed tomography (CT) and magnetic resonance (MR) scans, several new applications for stereotactic surgery were introduced. They included biopsy of intracranial lesions, tumor biopsy, placement of radiation sources into intracranial tumors and aspiration of cysts and abscesses.[3] Furthermore, with the capability of using computer technology to plan trajectories, its use was extended to open craniotomy procedures.[9] Although the systems were accurate, easy to use and provided stable probe-holders, there were some drawbacks. They included the need to attach head pins prior to obtaining scans for the procedure, bulkiness of the frame, obstruction of the operative field by components of the frame and the inability to navigate the operative field as the operation progressed. To overcome some of these problems, frameless stereotactic systems were introduced.[1,4,5,11,12,14,16,18] They quickly became popular because they allowed the use of pre-operative images for virtual planning, they prevented unhindered access to the operative site during craniotomy procedures and provided the ability to navigate the operative field with real-time localization of position on CT and/or MR images. Frameless stereotaxy, however, also has its own shortcomings. These include difficulties in moving the aiming probe in precise intervals relative to a pin-point target, adjusting the trajectory while maintaining the aim on a target and carrying out a dissection along the path of a probe placed at the target due to obstruction by the probe-holder. To overcome these difficulties, the author has used a modified Patil stereotactic frame as an instrument holder for the frameless stereotactic system. In this paper, a technique to use a modified frame in combination with a frameless system is described.

## METHODS

### The Frameless Stereotactic System

The BrainLab vector vision neuronavigation system (BrainLAB AG, Feldkirchen, Germany) was used in combination with a frame-based custom-designed stereotactic system for the procedure.

**Figure 1:**
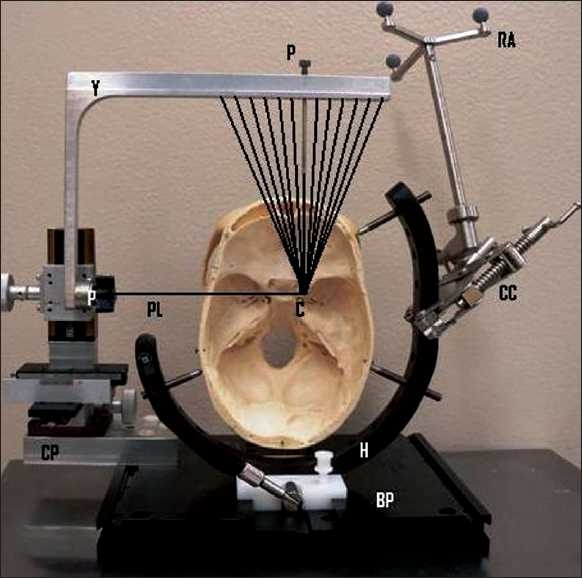
(Y = yoke; P = probe-holder; RA = reference array; PL = pivot-line; C = center of the arc; P = pivot; CC = c-clamp; CP = coordinate platform; H = head holder; BP = base plate). The lines converging from the yoke to the center of the arc represent the trajectories of the holes in the horizontal arm of the yoke

The stereotactic frame is a modified Patil system[13] that was custom made at our facility. The system consists [Figure 1] of a base plate to which the head-holder (with a three-point fixation system) is attached. On one side of the base plate is a coordinate platform that carries the X-Y-Z rack and pinion type of coordinate adjuster. A single armed yoke is attached to the coordinate platform by means of a pivot. The horizontal arm of the yoke has 13 holes through its vertical thickness, which serve to hold the probe holder. The middle hole is at 90 degrees to the horizontal surface of the yoke. The center of the arc of the system is at a point where the pivot-line (a line perpendicular to the center of the pivot) intersects a perpendicular line through the center of the middle hole. All other holes are placed on either side of the middle hole at 5-degree increments (up to 30 degrees) in relationship to the center of arc. The diameter of the holes is equal to the outer diameter of a 12-gauge needle. The radius of the arc of the frame as measured from the top surface of the yoke is 15.5 cm. Because the top surface of the yoke is flat, this distance is true only for the middle hole. The other holes have a slightly greater distance. These distances are pre-calculated for each hole and correction is made for it during the procedure. The system also has a probe-holder with an inner diameter equal to the outer diameter of a 14-gauge probe that can snugly fit into the holes.

### The Procedure

Over the past 2 years, this method was used in 10 procedures. Seven of these procedures were for placement of depth electrodes for seizure activity recording, six for the amygdalohippocampal complex and one for the supplementary motor cortex. Three procedures were for entry into the temporal horn through the middle temporal gyrus during seizure surgery.

Pre-operatively, MR and CT images were obtained using the standard protocol for the frameless system. Image fusion of both these modalities were performed and then loaded onto the computer used for navigation.

The procedure was performed under general anesthesia. The patient’s head was firmly fixed in the stereotactic head-holder with three head-pins. The reference array was attached to the head-holder by means of a c-clamp. To secure it in place tightly, sand paper was interposed between the clamp and the head-holder. Facial surface registration was performed using a z-touch laser pointer (BrainLAB AG). In addition, vertex and occipital area registration were performed using the pointer. The registration was recorded through the infrared camera. To improve the accuracy, both sides of the face were included in the registration. After good accuracy of the system was confirmed, the procedure was started. The cranial opening was made. The yoke was attached to the pivot and the pointer was inserted through the middle hole on the yoke [Figure 2a]. Because the pointer tip was tapered and its proximal end was greater in diameter than the holes in the yoke, it could not be fully inserted through the hole. This kept the pointer-tip outside the head. The distance from the pointer tip to the pivot line was calculated by measuring the distance from the pointer tip (with it maximally inserted) to the top surface of the horizontal surface of the yoke and subtracting it from the radius of the arc. The tool-tip offset mode on the BrainLab monitor screen was used to set this as the offset distance so that although the pointer-tip was outside the head, the tip of the probe-line on the monitor represented the center of the arc. Next, the center of the arc was brought on the target [Figure 2b] using the X-Y-Z adjuster (under the drape). The yoke was then rotated on the pivot while viewing the monitor to choose the best plane. When needed, adjustments were made in the coronal plane by changing the hole of approach on the yoke. The pointer was removed and the probe holder was inserted. Through this probe holder, depth electrodes [Figure 2c] or biopsy probe were inserted to the target. The depth of insertion was equal to the radius of arc of the frame (15.5cm) plus corrections for the angle of approach in the coronal plane and the length of the probe-holder above the surface of the yoke. In the procedures where the system was used to gain access to the temporal horn through the middle temporal gyrus, the biopsy probe was used as guide to the target while advancing the dissection to it. When required, the pointer could be used in a free-handed, standard fashion. When depth electrodes were placed at the target, post-operative scans were obtained to confirm accurate electrode placement.

**Figure 2a:**
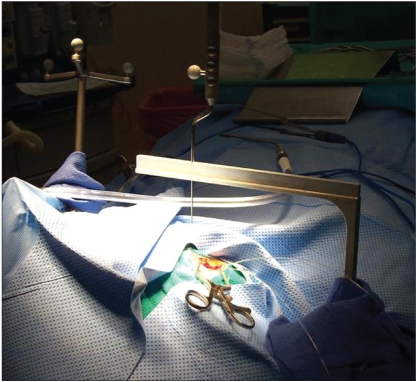
Photograph of the procedure. The reference array is visible at the left upper corner of the picture. The pointer is in the middle hole on the yoke

**Figure 2b:**
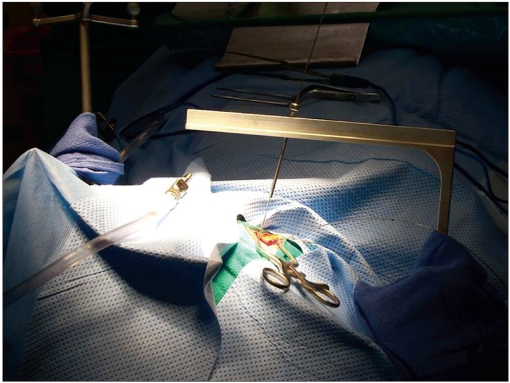
These images on the navigation system’s monitor were obtained after the center of the arc was brought on the target using the X-Y-Z adjuster. The outer cross-mark represents the position of the pointer tip while the deeper cross-mark represents the position of the center of the arc of the stereotactic frame

**Figure 2c:**
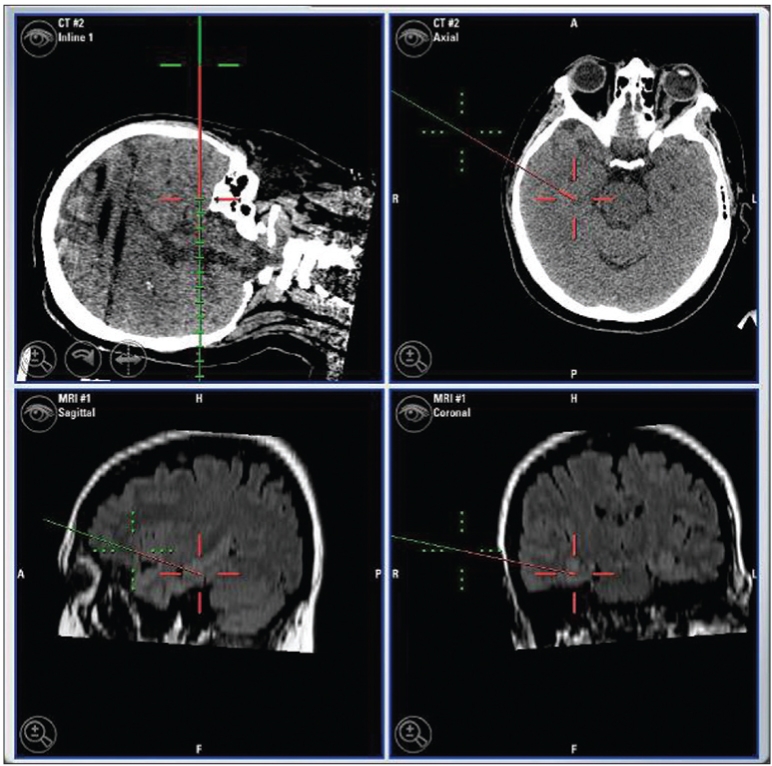
The probe-holder is aimed at the target and the depth electrode is inserted into the target

## RESULTS

There were no complications from the procedure. Post-operative scans showed the electrodes to be within 2.5 mm of the planned target in all three planes. During open craniotomy, there was very little obstruction of the view by the probe holder and the temporal horn was reached easily and accurately.

## DISCUSSION

Although a frameless system has the distinct advantage of being able to quickly navigate the surgical site and provide information about the site of surgical action on CT and/or MR images, this capability can also become a disadvantage because the location of the pointer tip can quickly come off target as the surgeon’s eyes move away from the operative site to the monitor. In addition, when there is the need to place a probe at a very small target, a stabilizing device is necessary. There are different types of probe-stabilizing attachments available for use with a frameless system. One type of system has articulated arms[15,17] that can be locked. These also have an associated guide that allows rotation and adjustment of the probe-holder. In this type of system, the head is fixed rigidly to the table and the stabilizing arm is attached to the table via the head-holder clamp. Another type of device is the skull-mounted system,[2,6,7,10] which is directly attached to the skull and does not need an articulated arm. However, none of these systems have the capability to smoothly move the pointer in precise intervals relative to a pin-point target. Furthermore, none of these are a center of the arc system. It is difficult to change the trajectory after target acquisition, without losing track of the target. Furthermore, in stereotactic craniotomies, the surgeon’s view during dissection to a deep target can be obstructed because of the size of traditional probe-holders. To overcome these drawbacks, the author has used a modified stereotactic frame to act as an instrument holder for the frameless stereotactic systems.

In the author’s system, the pointer of the frameless system is securely held in place and its position is adjusted by rack and pinion movement on the frame. The probe-holder can therefore be moved discreetly in three planes, enabling the pointer to precisely aim at small targets with ease. In addition, because it is a center of the arc system, the trajectory can be changed in both the sagittal and the coronal planes without altering the aim of the pointer on the target. The yoke that holds the probe-holder is extremely thin, thereby minimizing the obstruction of the surgeon’s view. The reduction in weight also minimizes the potential sag that is inherent in a single-armed yoke design. To further reduce the weight the probe holding holes are within the yoke, with their trajectories centered around a point on the pivot line. This point, thus, forms the center of a virtual arc created by the positions of the holes. The system was designed with only one coordinate platform to improve freedom of movement of the yoke.

Conventional frames are difficult to use during open craniotomy procedures because they are bulky and cumbersome. The modified frame described in this paper solves this problem because the yoke of the frame (which is very thin) is the only hardware in the operative field. This is evident in Figure 2 a and c. In addition, the yoke is less bulky than the biopsy arm and other associated probe-adjustment devices of the frameless systems. Furthermore, because the yoke can be rotated in and out of the field (around the pivot) and the trajectory can be changed without the need to reposition the pointer on the target, it is much easier to use than the usual accessories of the frameless systems.

The system has the following additional advantages: (1) pre-operative images can be obtained without the frame, (2) coordinates can be adjusted by simply identifying the target on the monitor and moving the center of the arc on it, without the need to measure or calculate the coordinates, (3) dynamic trajectory planning can be done by moving the arc and viewing the structures on CT and/or MR images plane by plane. The combination method does not preclude the use of a frameless system by itself. The pointer can, therefore, also be used free-handedly to navigate the operative site. The current technique is best suited for placement of electrodes or probe (to act as a guide during dissection toward a deep target or for biopsy purpose) into deep structures. Therefore, for other applications such as tumor resection, the free-handed technique will still be necessary. The author has used the current system in only 10 procedures over the last 2 years, because it was used mainly for placement of probes or electrodes into deep structures for which sub-millimeter accuracy was not as critical.

The current technique is basically frameless stereotaxis. Its accuracy therefore will be as good as the accuracy of the frameless system. This method has the disadvantage of introducing inaccuracies if the reference array moves or if surface registration is inadequate. The stability and the attachment of the reference array clamp to the head-holder can be improved by interposing sandpaper between them. The registration process can be improved by performing a z-touch laser beam registration on both sides of the face and pointer registration on both sides of the vertex and occipital areas. Accuracy can also be improved by including CT images in the registration and navigation process.

In summary, this is a simple technique to combine the advantages of frame-based and frameless stereotactic systems. Its main advantages include the ability to precisely aim the pointer at the target with ease, maintain the aim of the pointer on the target while planning the trajectory and the ability to firmly hold a probe in place. This method is suited for biopsy of deep lesions, placement of deep electrodes into brain and open craniotomy approach to deep structures.

## References

[1] Barnett GH, Miller DW, Weisenberger J (1999). Frameless stereotaxy with scalp-applied fiducial markers for brain biopsy procedures: Experience in 218 cases. J Neurosurg.

[2] Gildenberg PI (1990). The history of stereotactic neurosurgery. Neurosurgery Clinic of North An.

[3] Gralla J, Nimsky C, Buchfelder M, Fahlbusch R, Ganslandt O (2003). Frameless stereotactic brain biopsy procedures using the Stealth Station: Indications, accuracy and results. Zentralbl Neurochir.

[4] Gumprecht HK, Widenka DC, Lumenta CB (1999). BrainLab Vector Vision Neuronavigation System: Technology and Clinical experience in 131 cases. Neurosurgery.

[5] Hall WA, Liu H, Truwit CL (2000). Navigus trajectory guide. Neurosurgery.

[6] Holloway KL, Gaede SE, Starr PA, Rosenow JM, Ramakrishnan V, Henderson JM (2005). Frameless stereotaxy using bone fiducial markers for deep brain stimulation. J Neurosurg.

[7] Horsley VA, Clarke RH (1908). The structure and functions of the cerebellum examined by a new method. Brain.

[8] Kelly PJ (1990). Stereotactic craniotomy. Neurosurgery Clinic of North An.

[9] McGirt MJ, Woodworth GF, Coon AL, Frazier JM, Amundson E, Garonzik I, Olivi A, Weingart JD (2005). Independent predictors of morbidity after image-guided stereotactic brain biopsy: A risk assessment of 270 cases. J Neurosurg.

[10] Martin AJ, Larson PS, Ostrem JL, Sootsman WK, Talke P, Weber OM, Levesque N, Myers J, Starr PA (2005). Placement of deep brain stimulator electrodes using real-time high-field interventional magnetic resonance imaging. Magn Reson Med.

[11] Paleologos TS, Dorward NL, Wadley JP, Thomas DG (2001). Clinical validation of true frameless stereotactic biopsy: Analysis of the first 125 consecutive cases. Neurosurgery.

[12] Patil AA (1984). Computed tomography (CT) plane of the target approach in CT stereotaxis. Neurosurg.

[13] Price R (2003). The advantages of frameless stereotactic biopsy over frame-based biopsy. Br J Neurosurg.

[14] René L (2002). Bernays and Wernholt von Tempelhoff, “Snapper-Stereoguide” – A New Device for Image Guided, Frameless Stereotactic Procedures in the Open MR. Medical Laser Application.

[15] Ringel, Florian M.D, Ingerl, Dominik M.D, Ott, Stephanie M.D, Meyer, Bernhard M.D (2009). Varioguide: A New Frameless Image-Guided Stereotactic System-Accuracy Study and Clinical Assessment. Neurosurgery.

[16] Smith JS, Quiñones-Hinojosa A, Barbaro NM, McDermott MW (2005). Frame-based stereotactic biopsy remains an important diagnostic tool with distinct advantages over frameless stereotactic biopsy. J Neurooncol.

[17] Vogele M, Bale RJ Targeting device (Medtronic, VERTEK^™^) European Patent 0871407.

[18] Woodworth GF, McGirt MJ, Samdani A, Garonzik I, Olivi A, Weingart JD (2006). Frameless image-guided stereotactic brain biopsy procedure: Diagnostic yield, surgical morbidity, and comparison with the frame-based technique. J Neurosurg.

